# The cost-effectiveness challenge: is it worth it?

**DOI:** 10.1186/s13195-015-0095-4

**Published:** 2015-01-27

**Authors:** Martin Knapp

**Affiliations:** Personal Social Services Research Unit, London School of Economics and Political Science, London, WC2A 2AE UK

## Abstract

Scarcity of resources means that difficult choices have to be made about how to use them. Cost-effectiveness evidence provides a way to help decision-makers get ‘best value’ from their resources when choosing between two or more clinical or other interventions. Often it is found that one intervention has better outcomes than another, but also costs more. In these circumstances there is a need for the decision-maker to reach a view as to whether those better outcomes are ‘worth’ the higher costs, necessitating difficult trade-offs. Illustrations from the dementia field are given to illustrate how these trade-offs might be made. For strategic decisions it has often proved helpful to use a generic outcome measure such as the quality-adjusted life year. The fundamental aim of a healthcare system is not to save money, but to save and improve lives. Cost-effectiveness and similar analyses can help by showing how to get the most out of available resources.

## Introduction

There are never enough resources to meet everyone’s health or other needs, or to satisfy everyone’s wants. Consequently, anyone taking decisions on how to spend available resources faces some tough choices. Payers, hospital managers and other healthcare decision-makers generally want to use their budgets to achieve the best outcomes they can: meeting patients’ needs, reducing symptoms and improving quality of life. But converting available resources into best outcomes is no easy feat.

Increasingly, these decision-makers are turning to cost-effectiveness evidence to help them. Cost-effectiveness analyses compare the costs and outcomes of two or more clinical or other interventions to try to get ‘best value’.

## Examining cost-effectiveness

The researcher - usually an economist working with clinical colleagues - would first add up the costs associated with each intervention (the treatment itself plus other care and support services used by patients) and subtract any downstream monetary savings (perhaps because people get healthier, use fewer services or avoid nursing home admission). The researcher would also measure the outcomes from each intervention, using the kind of symptom, functioning and quality of life scales familiar to clinical researchers.

The next part is the hardest: blending together the cost and outcome data. For example, consider the choice between two medications for treating dementia. If one is both cheaper and more effective than the other, it would immediately look attractive to the hard-pressed budget-holder because it improves health or wellbeing while simultaneously saving money. In such circumstances, in economics parlance one medication dominates the other: it is obviously more cost-effective. But the more cost-effective option might not always get chosen, for there may be other considerations, such as fairness, availability and patient preferences.

## Is it worth it?

Complications arise when one medication has better health outcomes but higher costs (for the medication plus supporting care) than the other. The quandary is whether the better outcomes justify the higher cost. Whilst we would all like to see better outcomes for dementia patients, we must remember that resources are finite, and so committing extra resources to treating one patient will inevitably mean fewer resources for other patients.

Judging whether the outcome difference justifies the higher cost is not straightforward; indeed, it is a value judgement. Someone has to look at the trade-off between better outcome and higher cost and ask ‘Is it worth it?’ We all make these kinds of judgements in our everyday lives: an option that pleases us most (has the best outcomes) might be more expensive in time or money than the alternatives, but we might choose it because we think it is ‘worth it’.

An intervention can therefore be cost-effective even if it costs more. Consider an example. Evaluation of cognitive stimulation therapy in England found that patients with mild-to-moderate dementia receiving this therapy over 8 weeks had better cognitive and quality of life outcomes than people getting standard care [1], but costs were slightly higher. The economic analysis calculated the incremental cost-effectiveness ratios for two outcomes: £75 per 1-point improvement on the Mini Mental State Examination and £23 per 1-point difference on Quality of Life in Alzheimer's Disease [2]. Are these amounts worth paying? We do not know definitively because the answer depends on a decision-maker’s willingness to pay these amounts for these particular outcome gains.

## Quality-adjusted life years and thresholds

An additional complication comes when a strategic decision-maker must allocate resources between different clinical areas: outcome measures linked to symptoms will be disorder-specific and hence incomparable across disorders. In these cases, an additional generic (all-disorder) measure of outcome can be used: the quality-adjusted life year (QALY) shows the effects of treatment both in extending life and improving life quality. A commonly used tool is EQ-5D [3], which performs well across most disorders. A tool that is dementia-specific but nevertheless generates generic QALY measures stems from the DEMQOL [4,5].

In the START (STrAtegies for RelaTives) study, evaluating a coping strategy for family carers of dementia patients, QALYs were measured alongside clinical outcomes [6]. The intervention was effective over 8 months in improving carers’ mental health and health-related quality of life. The economic evaluation found that, *inter alia*, costs were slightly but not significantly higher for the intervention group compared with treatment as usual, and that the cost per additional QALY for carers was £6,000 [7].

Is that amount worth paying? Again, we do not immediately know the answer. However, we can now refer to a threshold value recommended by the health technology assessment body for England and Wales, the National Institute for Health and Care Excellence (NICE). NICE has a framework to help decide whether better outcomes are ‘worth’ the higher costs sometimes necessary to achieve them; decisions are taken by expert groups comprising clinicians, patients, researchers and the public [8]. NICE would consider that a medication or other intervention costing more than £20,000 per QALY is generally not ‘worth it’ since the resources could be better spent elsewhere in the healthcare system. The threshold is not a rigid rule [9], but provides guidance in making tough choices, and it reminds all of us - doctors, nurses, patients, carers, taxpayers, voters - that resources are scarce.

By reference to this threshold, the START intervention for dementia carers is certainly cost-effective over an 8-month period and, indeed, the 24-month results also look encouraging [10].

## Contributing to better health

This kind of analysis has become very common across all clinical and health services research literatures [11], although the number of cost-effectiveness studies in the dementia field remains modest [12]. It should be standard evaluative practice for any new intervention. If (say) a disease-modifying treatment for Alzheimer’s was to be developed, an economic evaluation would need to cost the diagnostic testing, treatment and other services, measure any savings from delayed care home admission, and combine these monetary data with evidence on gains in health, quality of life and life-span. These would need to be compared with costs, savings and outcomes for standard treatment and care to judge both relative effectiveness and value for money.

Economists work with clinical researchers to evaluate whether medications, psychosocial therapies, care arrangement, risk-reduction strategies or other interventions are not only effective but also cost-effective. Remember that the fundamental aim of a healthcare system is not to save money, but to save and improve lives. However, the best way to achieve this aim is to make best use of the resources that are available, which in turn means getting an understanding of cost-effectiveness and highlighting the trade-offs between better outcomes and higher costs that often have to be made.

## Abbreviations

NICE: National Institute for Health and Care Excellence
QALY: quality-adjusted life year
